# Aneurysmal bone cyst of the talus: a case report of local recurrence treated with adjuvant therapy

**DOI:** 10.1093/bjrcr/uaaf041

**Published:** 2025-07-28

**Authors:** Yasutaka Takagi, Hiroshi Yamada, Hidehumi Ebara, Hiroyuki Hayashi, Hiroyuki Inatani, Yuta Nakamura, Koichiro Ae, Aki Nakanami, Tetsutaro Yahata, Hiroyuki Tsuchiya

**Affiliations:** Department of Orthopaedic Surgery, Tonami General Hospital, Tonami, Toyama 939-1395, Japan; Department of Orthopaedic Surgery, Tonami General Hospital, Tonami, Toyama 939-1395, Japan; Department of Orthopaedic Surgery, Tonami General Hospital, Tonami, Toyama 939-1395, Japan; Department of Orthopaedic Surgery, Tonami General Hospital, Tonami, Toyama 939-1395, Japan; Department of Orthopaedic Surgery, Tonami General Hospital, Tonami, Toyama 939-1395, Japan; Department of Orthopaedic Surgery, Tonami General Hospital, Tonami, Toyama 939-1395, Japan; Department of Orthopaedic Surgery, Tonami General Hospital, Tonami, Toyama 939-1395, Japan; Department of Rehabilitation Medicine, Tonami General Hospital, Tonami, Toyama 939-1395, Japan; Department of Rehabilitation Medicine, Kanazawa University Hospital, Kanazawa, Ishikawa 920-8641, Japan; Department of Orthopaedic Surgery, Yokohama Sakae Kyosai Hospital, Sakae-ku, Yokohama, Kanagawa 247-8581, Japan

**Keywords:** aneurysmal bone cyst, adjuvant therapy, high-speed burr, phenol-ethanol ablation

## Abstract

Aneurysmal bone cyst (ABC) is a locally destructive benign tumour-like condition of the bones with blood-filled cystic cavities. The talus is an extremely rare site for an ABC, with <20 reported cases till 2012 based on a PubMed database search. Aneurysmal bone cyst recurrence in the talus after curettage and bone grafting is extremely rare. To the best of our knowledge, no detailed reports of resection and adjuvant therapy with artificial bone packing of recurrent ABC of the talus have been published. We report a case of ABC in the talus of a 9-year-old boy. As the initial surgery consisted of only lesion resection and artificial bone (beta-tricalcium phosphate [TCP]) packing, local recurrence was diagnosed. Reoperation was performed 5 months after the initial surgery. The recurrent lesion was resected using a curette, and the bone cavity septum was shaved with a high-speed burr. Phenol-ethanol ablation was used as an adjuvant with artificial bone (beta-TCP) packing to prevent recurrence. No local recurrence was observed 36 months after the reoperation. This extremely rare case of resection and adjuvant therapy with artificial bone packing of recurrent ABC of the talus highlights the need for careful observation to assess the progression of ankle joint osteoarthrosis.

## Introduction

Aneurysmal bone cyst (ABC) is a benign tumour-like condition of the bones with locally destructive blood-filled cystic cavities. The talus is an extremely rare site for an ABC.^1^^,^^2^ A PubMed database search revealed <20 ABC cases reported till 2012. Aneurysmal bone cyst recurrence of the talus after curettage and bone grafting is extremely rare.^3^^,^^4^ Here, we report a case of an ABC in the talus of a 9-year-old boy.

## Case presentation

The patient was a 9-year-old boy presenting with the chief complaint of left ankle joint pain and gait abnormality. The medical history was unremarkable. A year and a half ago, the patient starting experiencing left ankle joint pain with no obvious cause. Although he visited a nearby doctor, no abnormalities were detected on radiographic evaluation. He visited a doctor again due to pain and abnormal gait and was diagnosed with a bone tumour. Foot examination revealed no visible or palpable swelling, normal local temperature, and tenderness on deep palpation on the dorsal aspect of the ankle.

## Investigations

**Figure 1.**
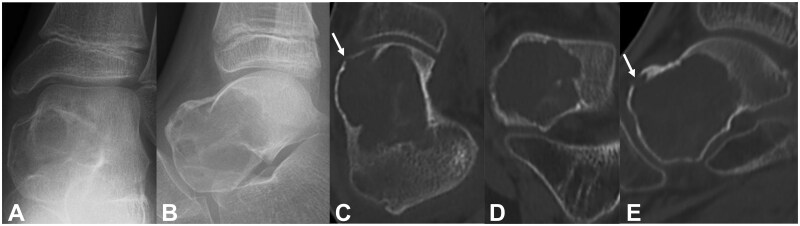
(A and B) An X-ray examination revealed a radiolucent lesion in the left talus. (C, D, and E) Bone radiolucency and cortical thinning were observed in the left talus. Findings suggestive of a pathologic fracture were observed (white arrow).

**Figure 2.**
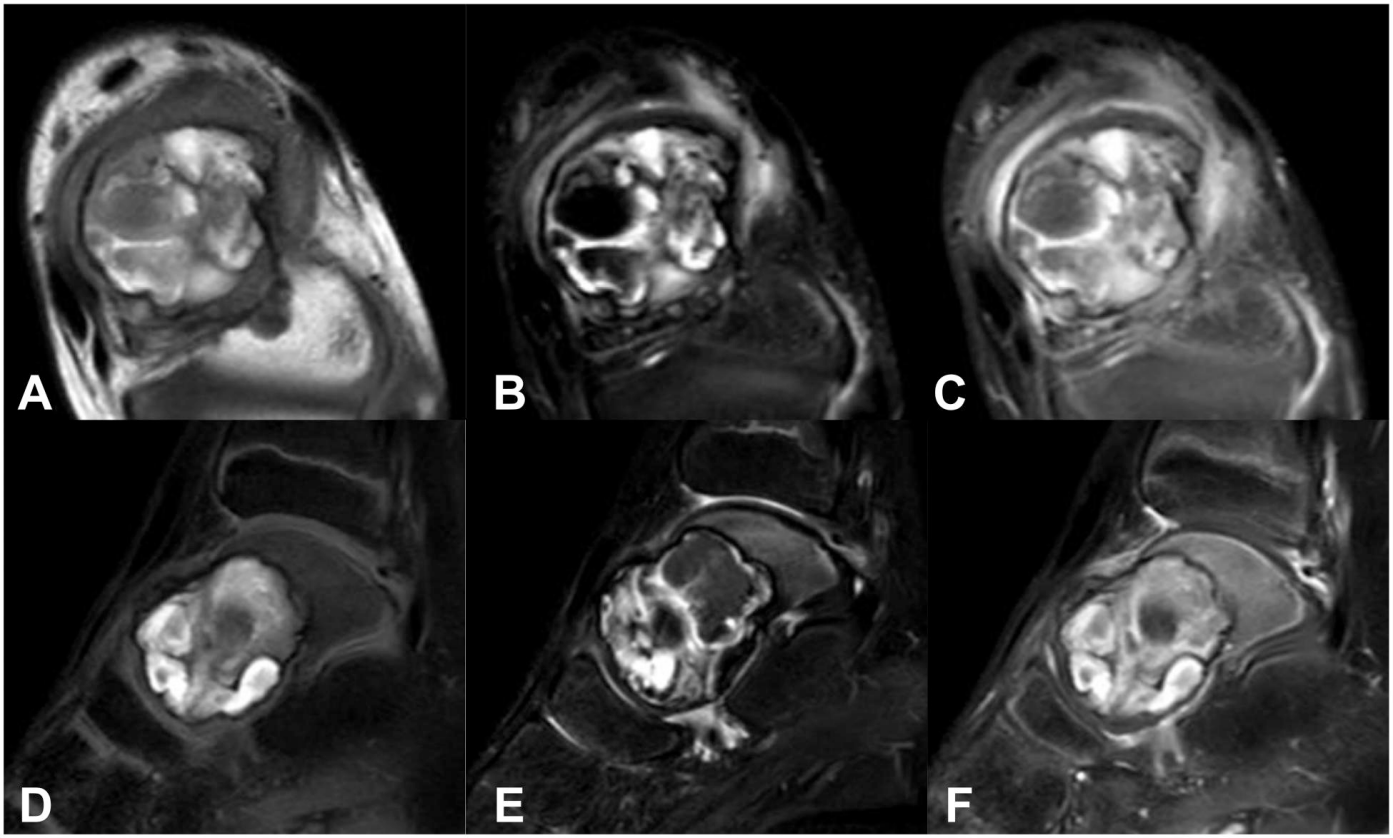
In the MRI examination, T1: Fat suppression revealed high signal, T2: Fat suppression revealed a mixture of low and high signals suggesting the presence of bleeding, and fat suppression on gadolinium contrast enhanced the peripheral area slightly ((A) T1 axial, (B) T2 fat suppression axial, (C) fat suppression on gadolinium contrast-enhanced axial, (D) T1 fat suppression sagittal, (E) T2 fat suppression sagittal, and (F) fat suppression on gadolinium-contrast-enhanced sagittal).

Radiographic examination revealed a radiolucent lesion on the left talus. Computed tomography (CT) revealed a radiolucent lesion and cortical thinning in the left talus. Findings suggestive of pathological fractures were observed (Figure 1). Magnetic resonance imaging (MRI) examination revealed a high signal on T1-weighted images (with fat suppression) and low and high signals on T2-weighted images (with fat suppression) suggestive of bleeding; furthermore, gadolinium contrast-enhanced imaging with fat suppression revealed the lesion margin (Figure 2).

## Treatment and outcome

**Figure 3.**
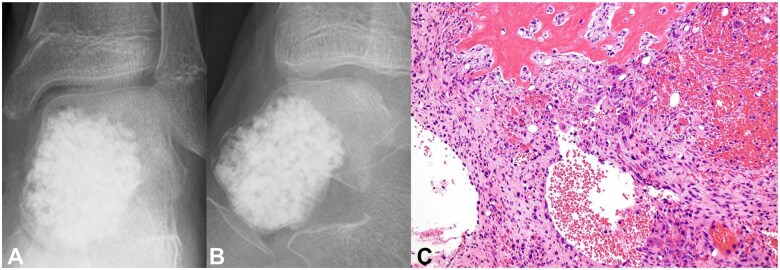
(A and B) Excision of the lesion and artificial bone (beta-TCP) packing were performed. (C) Permanent pathological examination confirmed the diagnosis of aneurysmal bone cyst.

**Figure 4.**
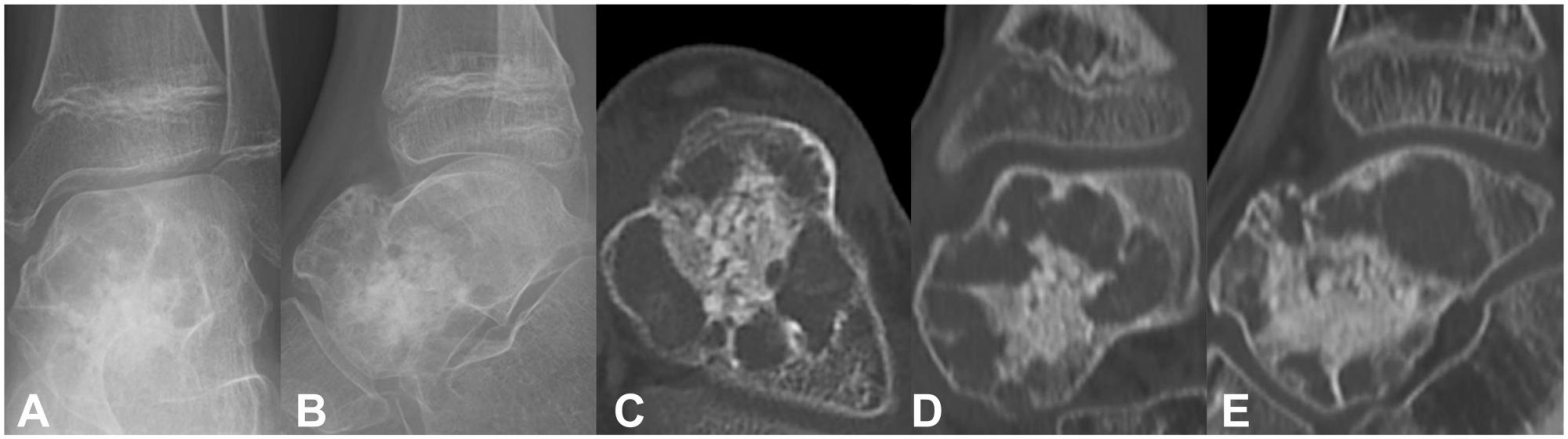
(A and B) Radiograph 4 months postoperatively revealed resorption of the artificial bone and bone translucency. (C, D, and E) CT examination 5 months postoperatively revealed a mixture of bone sclerosis and bone lucency.

**Figure 5.**
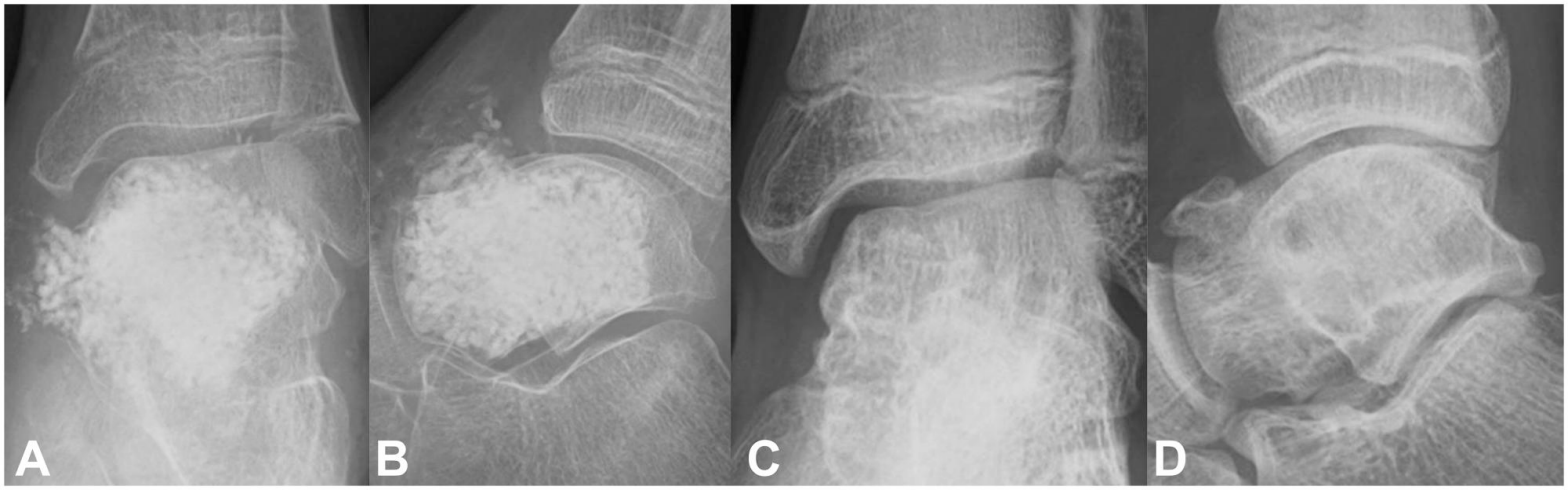
(A and B) The bone sclerosis site and remaining lesion were excised using a high-speed burr and phenol-ethanol ablation as adjuvant, and artificial bone (beta-TCP) packing was performed. CD: No local tumour recurrence was observed 36 months after the reoperation and the artificial bone (beta-TCP) was replaced with new bone.

A simple bone cyst or ABC was suspected, and during the initial surgery, lesion curettage and artificial bone (beta-TCP, AFFINOS [Kuraray Co., Tokyo, Japan]) packing were performed. Permanent pathological examination revealed multilocular cysts retaining blood, reactive bone sclerosis, and osteoclast-type multinucleated giant cells, leading to a definitive ABC diagnosis (Figure 3). Radiography 4 months postoperatively revealed artificial bone resorption and bone translucency. CT examination 5 months postoperatively revealed a mixture of osteosclerosis and bone translucency (Figure 4). Tumour recurrence was diagnosed and reoperation was performed. The bone sclerosis site and remaining lesion were resected using a curette, and the septum in the bone cavity was shaved using a high-speed burr. A gauze ball with phenol was placed in the bone cavity, followed by a gauze ball with 95% ethanol to neutralize the phenol. Distilled water was used to irrigate the bone cavity. These procedures were repeated 3 times. Phenol-ethanol ablation for opened cortical bone was performed using the same procedures. After the ablation, artificial bone (beta-TCP) was filled in the bone cavity (Figure 5). Subsequently, 36 months after the reoperation, no local tumour recurrence was observed, and the artificial bone (beta-TCP) was replaced with new bone (Figure 5). Moreover, 36 months postoperatively, the patient was pain-free with no restriction in the range of motion of the ankle and was participating in club activities as a sprinter.

## Discussion

In the context of ABC of the talus, Sharma et al^1^ reported that the ABC of the talus is a very rare lesion with <20 cases reported by 2012 using a PubMed search. The standard of care for ABC is curettage with or without bone graft, depending on the resultant void, with local recurrence being common. Therefore, various adjuvants, including cement, high-speed burr, argon beam, phenol, and cryotherapy, have been developed to reduce recurrence. After intralesional resection of an ABC, a high-speed burr can be used to augment curettage by mechanical disruption of the circumscribing bone level. In a case series of 40 patients, Gibbs et al^5^ reported local control rates of approximately 90% after a median 7.2-year follow-up of treatment using curettage and high-speed burr without the use of liquid nitrogen, phenol, or other adjuvants. Dormans et al^6^ reported that their surgical technique using a high-speed burr resulted in an 82% cure rate. Wang et al^7^ concluded that a high-speed burr with curettage and bone graft was a reasonable approach for ABC treatment as recurrence was observed in only 1 of the 31 patients included in their study. However, a high-speed burr has not been reported to decrease local recurrence in all studies. In a retrospective comparative study, Lin et al^8^ reported no effect in the 5-year disease-free survival associated with the use of a high-speed burr. Phenol, also known as carbolic acid, is produced in mass quantities from petroleum and is a precursor of various materials, including plastics, pharmaceuticals, and analgesics. In ABC treatment, phenols have been used to sterilize or wash the lesion and remove remaining neoplastic cells following curettage. In a retrospective case series, Capanna et al^9^ reported a 7% recurrence rate following curettage and phenol treatment versus 41% with curettage alone. Bitzan et al^10^ reported that curettage and phenol therapy employed in nine patients resulted in no recurrences.

Based on our review of the literature, no detailed reports of resection and adjuvant therapy for recurrent ABC cases of the talus have been reported previously. In this case, as the initial surgery included only lesion resection and artificial bone (beta-TCP) packing, local recurrence was diagnosed. Reoperation was performed and the resected site was treated using a high-speed burr and phenol-ethanol ablation as adjuvant with artificial bone (beta-TCP) packing. No local recurrence was observed 36 months after the reoperation. This report highlights the need of careful observation for assessing the progression of ankle joint osteoarthrosis.

## Learning points

- We report an extremely rare case of resection of a recurrent ABC of the talus and adjuvant therapy with artificial bone packing.
- Complete lesion resection using a high-speed burr and phenol-ethanol ablation as adjuvant was needed to prevent local recurrence.

## Data Availability

Medical imaging data will not be shared because it is not fully anonymous.
